# Glossophobia: connections to some psychological and demographic factors among university students

**DOI:** 10.3389/fpsyt.2026.1883250

**Published:** 2026-10-30

**Authors:** Yossef M. Eid, Sohil Alqazlan

**Affiliations:** 1 Department of Psychology, College of Humanities and Administrative Studies, Onaizah Colleges, Onaizah, Saudi Arabia; 2 Special Education Department, College of Education, Imam Mohammad Ibn Saud Islamic University (IMSIU), Riyadh, Saudi Arabia

**Keywords:** age, gender, motivation, public speaking anxiety, self-efficacy, university students

## Abstract

Glossophobia is a condition that negatively impacts a university student’s ability to speak in front of an audience. This study examined the connections among college students’ motivation, self-efficacy, and glossophobia. A quantitative cross-sectional correlational and comparative design was used with 126 university students. As data-gathering tools, the scales used were the Glossophobia Scale, Self-Efficacy Scale, and Motivation Scale. The findings showed significant negative associations between glossophobia and both self-efficacy and motivation. No significant association was found between age and glossophobia. No significant gender differences were found in glossophobia. The investigation results show that most students are frightened of being teased and laughed at. They also worry that speaking in front of an audience may repeat earlier bad experiences.

## 1 Introduction

Glossophobia manifests as anxiety during public speaking, affecting students’ willingness to participate in class discussions and presentations. As stress and glossophobia increase, students’ learning attitudes and academic performance tend to decline (1). According to Herumurti et al. (2), glossophobia is a psychological condition that makes a person hesitant to speak in front of others. The individual felt he appeared silly and turned red as a result. The illness caused physical symptoms like sweating, numb hands, and blurry vision. The incident stemmed from a prior embarrassing public-speaking experience.

Glossophobia, or the dread of speaking in public, is a widespread phobia that affects millions of people worldwide. It is believed that over 75% of people suffer some amount of anxiety while speaking in public, whereas roughly 10% of people experience considerable anxiety and delaying behaviours connected to public speaking (3). Glossophobia may have a substantial influence on an individual’s work and personal life, since it can impair communication and limit prospects for growth. (4). Glossophobia, or speech anxiety as it is more commonly known, can negatively impact a student’s academic performance in college. Pratiwi et al. (5) found that 77.4% of the students in their study struggled to speak in front of others. In a separate study, Eid (6) discovered that 19% of male students in the Kingdom of Saudi Arabia suffer from a speech anxiety disorder. The findings of Bartholomay and Houlihan (7), who discovered that one in five people experience public speaking anxiety (PSA), are comparable. They continued by stating that one of the most common types of anxiety, PSA, causes excessive psychological and physiological reactivity.

Additionally, Glossophobia is primarily caused by poor preparation, fear of criticism, fear of failure, and perfectionism. These variables cause physical symptoms such as hand shaking, voice trembling, elevated heart rate, perspiration, and shortness of breath, which drastically reduce students’ public speaking ability (8). According to Hancock et al. (9) and Waheed (10), People with public speaking anxiety (PSA), a kind of social phobia, avoid speaking, especially in public, out of a sense of uneasiness or shame. It has a detrimental effect on the person’s way of life.

Self-efficacy corresponds to one’s confidence in one’s capacity to complete a task or achieve a desired outcome. According to Bandura (11), self-efficacy is “people’s self-reliance on their abilities to take specific initiatives or fully utilise their capabilities and thus achieve specific outcomes.” Bandura studied people who had phobias and introduced this term. Mastery experiences, Vicarious experience, Verbal persuasion, and Emotional state are the four main sources of self-efficacy (12).

If someone believes they are incapable of anything, it will negatively affect their perception of their ability to complete related tasks. On the other hand, a person with high self-efficacy will accept a challenge if they believe they can achieve it (13). According to Chamorro-Premuzic (14), motivation is “an internal state that drives behaviour and is directed toward the satisfaction of both instinctual needs and cultural needs and goals. It is dynamic rather than static in nature”.

This study examines glossophobia and its effects on college students’ capacity to fully engage in educational activities. It may be possible to learn more about why some students experience high levels of glossophobia and how to support them by examining its effects on students’ motivation to learn and sense of self-efficacy. However, limited research has examined the relationships among glossophobia, self-efficacy, and motivation simultaneously among university students in Saudi Arabia. Therefore, this study aimed to examine these relationships within the Saudi higher education context, providing context-specific evidence to inform academic counselling and support services.

### 1.1 Literature review

Glossophobia is the dread of speaking in front of an audience (in Greek, glossa means tongue, and Phobos means fear) (9). Glossophobia, or speech anxiety, is the dread of speaking in front of an audience. It can also manifest as a person’s cognitive nervousness when speaking with others. (15). In the literature, speech phobia is also known as communication phobia, communication apprehension (CA), and communication avoidance. (16). Glossophobia, or speech anxiety, is the fear of speaking in front of people. It can also manifest as a person’s cognitive nervousness when speaking with others. Public speaking anxiety is common for the average person. This is especially true for most college students (17). In the present study, glossophobia and public speaking anxiety are used interchangeably to refer specifically to anxiety associated with public speaking, without implying a clinical diagnosis of social anxiety disorder.

Symptoms of glossophobia include increased heart rate, tremors, blurred vision, memory loss, and dry mouth. Those with glossophobia will do everything they can to avoid going to many social occasions that will make them anxious. When the social event is unavoidable, they feeling of extreme anxiety and stress will be experienced by them (Hau 18). Individuals who develop a phobia of public speaking typically find it challenging to engage in daily activities, which can disable them and lower their quality of life. Sometimes signs of mental health issues might also be caused by such anxiousness. (19). These characteristics are visually summarised in **Figure 1** to provide a concise overview of the main manifestations of glossophobia.

**Figure 1:**
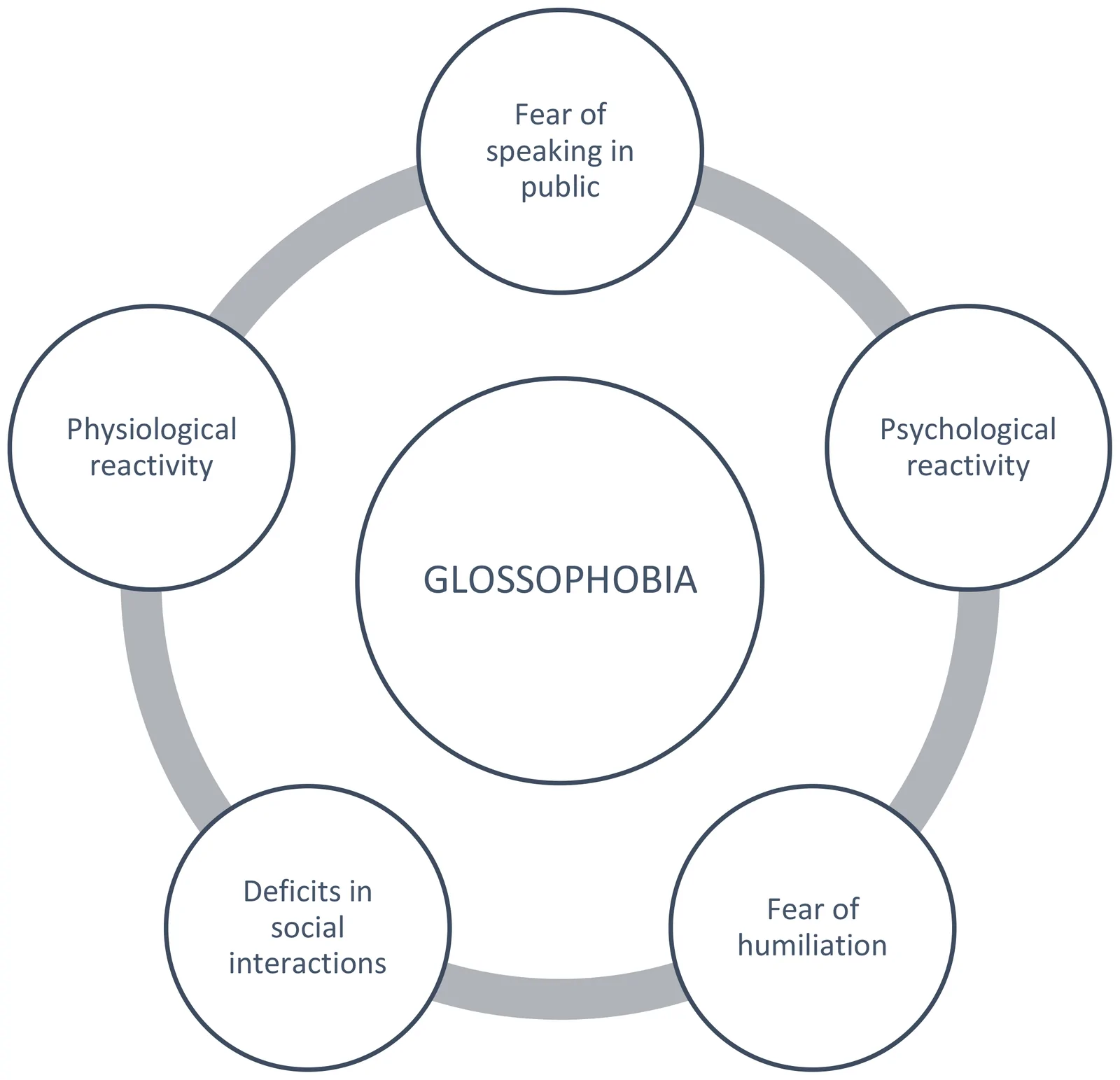
Characteristics of glossophobia (prepared by the author).

Anxiety is a frequent emotional reaction to stressful events, such as public speaking. Individuals suffering from anxiety disorders are more prone to suffer from glossophobia than those who do not suffer from this disorder (3). In fact, anxiety is one of the most important predictors of glossophobia, with those who have a lot of worry being more likely to have a lot of anxiety and avoidance behaviours when it comes to public speaking (4). Everyone feels that the tension that results from public speaking is a type of communication anxiety. According to Goberman et al. (20), some people experience adverse effects from this anxiety on their ability to communicate with others, concentrate in class, and advance their careers. According to Kamridah (21), there is a significant inverse relationship between speaking in public and the level of anxiety. When given a chance to speak in front of an audience, up to 40% of the population reports feeling anxious. However, the human disorder known as speech anxiety can be identified and treated by those who experience it (22). PSA is a type of state-based anxiety. Trait-based PSA is thought to be present in students who experience anxiety primarily related to the prospect of speaking in front of an audience. State-based anxiety is defined as students who expressly experience an increase or decrease in anxiety during a particular phase of the general speech process. These worries may go without warning or occur concurrently (23).

Hau Tse (18) stated that glossophobia is common among students and that students may fail in school due to anxiety. Several studies have been conducted to measure public speaking anxiety in university students. Khan et al. (24) and Perveen et al. (25) found that female students experience more significant anxiety in public speaking than male students. Similarly, Waheed’s (10) study concluded that there were significantly higher mean scores for female participants in the social phobia inventory, while Hancock et al. (9) found that more females (27%) had public speaking anxiety than males (14%). These findings underline that female students may be more prone to glossophobia than male students. However, according to Hau Tse (18), the theory that women experience more anxiety than men has not received enough support. Another interesting finding is that in the study by Khan et al. (24), fifth-year students showed the lowest level of anxiety, while Gaibani and Elmenfi (26) found a significant negative influence of age on public speaking anxiety. Glossophobia is more common in younger people than in older people, according to research (3).

Additionally, research shows that the audience’s makeup significantly increases anxiety. Hau Tse (18) asserts that the audience significantly impacts many physiological and psychological manifestations of anxiety related to public speaking. This is in line with the notion that people are typically more at ease around those they know well. Students who have abnormally high levels of public speaking anxiety may later behave in an avoidant, overt manner. Previous research has indicated that public speaking anxiety can be managed through various psychological and behavioural approaches. Nelson et al. (27) argued that public speaking anxiety is not necessarily something to be cured but rather a condition that can be managed. Similarly, Lihui (28) examined approaches combining psychological techniques with speech preparation and reported reductions in students’ public speaking anxiety. More recently, Fauzi et al. (29) highlighted strategies such as confidence-building seminars, tailored coaching, and interventions that address societal perceptions and cultural expectations as potential approaches to reducing public speaking anxiety. Beyond these management approaches, understanding the psychological factors associated with public speaking anxiety is also important, particularly self-efficacy and motivation.

According to Pratiwi et al. (5), self-instruction therapy combined with group counselling effectively lowered students’ glossophobia levels. Students’ motivation is crucial in determining their success (30). Recent research in Saudi Arabia has also examined learning motivation among Saudi students, highlighting the importance of understanding motivational processes within educational settings (31). Students’ motivation can be influenced by how they define “knowledge” and “learning.” In contrast to students who consider knowledge as a quantity that may alter and increase, those who define knowledge as a fixed quantity that a person has or does not have are less likely to be motivated to learn (32).

Emotions are closely related to both good and bad internal factors, like anxiety. Anxiety and other harmful elements can be significantly reduced and controlled when emotions are correctly identified. Even in a classroom setting, our emotions can significantly impact how we behave (33). Student-faculty relationships are often interpersonal, and interpersonal relationships are strongly correlated with the development and maintenance of emotions. When negative emotions are evoked, students are less motivated to approach the teacher and participate in class (34).

Both successful and unsuccessful experiences shape one’s sense of self-worth, which may contribute to the development of glossophobia. According to Bandura (11), there are two types of experiences: direct and alternative. Individuals rely on direct experiences related to their competence, abilities, and capabilities. Then he moves on to following up on other people’s successes, which is referred to as having “alternative experiences.” These experiences generate many expectations that support self-efficacy in social and academic areas and increase the individual’s willingness to make an extra effort to improve personal performance. According to Eid (35), personal achievements, particularly successful social interaction, are among the factors that have the most significant influence on self-efficacy. They are genuine experiences gained due to the individual’s interaction with the outside world.

Previous studies have demonstrated a strong correlation between self-efficacy and the severity of glossophobia. College students’ self-efficacy and English as a Foreign Language (EFL) speaking anxiety were compared by Qurnia and Marlina (36). The 63 study subjects were pupils in the Public Speaking course. The findings revealed a relationship between speech anxiety and self-efficacy in EFL students, and they explored the issue of gender. Prentiss (37) found that the best practices for reducing speaking anxiety include boosting confidence and learning valuable cross-disciplinary skills that promote self-efficacy. To summarise, numerous studies have shown that glossophobia is a common disorder and that many students are averse to speaking in front of others (5, 7, 18, 38, 39).

### 1.2 Current study

As part of their academic responsibilities, undergrads in particular are sometimes required to deliver presentations or talks. As a result, glossophobia and self-efficacy might have serious consequences for their academic progress and general well-being. The current study investigated self-efficacy, motivation, and glossophobia among university students in the Kingdom of the current study.

Raising awareness of glossophobia among college students is crucial for effectively addressing this fear, particularly when it intersects with low levels of motivation and self-efficacy. Understanding the intricate relationships between glossophobia, motivation, and self-efficacy can inform the creation of targeted counselling strategies aimed at enhancing self-efficacy to alleviate glossophobia. Increased self-efficacy empowers individuals to face their fear of public speaking with greater confidence and resilience. Moreover, addressing motivational factors can foster a more positive attitude toward communication challenges. It is essential to integrate these insights into educational programs to better support students. Providing workshops and resources focused on overcoming glossophobia can improve overall academic and social performance. Ultimately, a comprehensive approach that combines psychological support with skill development may be associated with more effective management of glossophobia and related anxieties.

### 1.3 Theoretical framework and conceptual model

To address the limitations of treating psychological constructs in isolation, this study adopts Bandura’s Social Cognitive Theory (SCT) as its overarching theoretical framework. According to SCT, human functioning is product of a triadic reciprocal causation involving cognitive, behavioral, and environmental influences. Within this framework, self-efficacy (cognitive self-appraisal) serves as a core self-regulatory mechanism that dictates how individuals perceive their capabilities, which in turn influences both their emotional states and behavioral drives.

In the context of public speaking, a student’s level of self-efficacy acts as a primary cognitive filter. When faced with a public presentation, students with low self-efficacy are more likely to engage in cognitive catastrophizing and anticipate failure, which directly intensifies somatic and emotional arousal, manifesting as glossophobia (speaking anxiety). Conversely, high self-efficacy fosters psychological resilience, shielding students from debilitating anxiety. Furthermore, SCT posits that cognitive self-appraisals directly govern motivation (behavioural drive). Students who judge themselves as highly capable (high self-efficacy) are highly motivated to engage actively in communication tasks, seeing them as opportunities for mastery rather than threats. In contrast, those experiencing high glossophobia experience a severe drop in motivation, retreating into avoidant behaviors to escape perceived social embarrassment.

Based on this integrated conceptual model, we hypothesize the following:

- Hypothesis 1 (H1): Self-efficacy will significantly and negatively predict glossophobia among university students.
- Hypothesis 2 (H2): Self-efficacy will significantly and positively predict students’ academic motivation.
- Hypothesis 3 (H3): Glossophobia will significantly and negatively predict academic motivation, acting as an emotional barrier to behavioural engagement.

### 1.4 Research questions

These are the research questions that the study was guided by.

1. How common is speech anxiety disorder (glossophobia) among university students in the Kingdom of Saudi Arabia?
2. To what extent do cognitive self-appraisals (self-efficacy) and academic motivation predict glossophobia levels among university students when accounting for demographic variables?
3. Does the integrated conceptual model based on Social Cognitive Theory effectively explain the interaction between students’ emotional arousal and behavioural drive?
4. Are there significant differences in glossophobia levels attributable to gender or age within the Saudi academic context?

## 2 Methodology

### 2.1 Study design

This study employed a quantitative cross-sectional correlational and comparative design. Data were collected using the Glossophobia Scale, Self-Efficacy Scale, and Motivation Scale. Appropriate statistical methods were used to analyse the collected data and examine the relationships among the study variables and differences across demographic groups. The authors confirm that the study was conducted in full compliance with academic integrity and ethical research standards.

### 2.2 Participants

The current study sample included 126 university students (*M* = 21.8 years, *SD* = 0.780; Range = 20–23). The participants were 64 male and 62 female students enrolled at the College of Education in the Kingdom of Saudi Arabia, providing a specific academic and cultural context. Participants were selected from eligible students enrolled at the College of Education during the study period using a random sampling approach, as described in the original study protocol. Each eligible student had an equal opportunity to be selected. Participation was voluntary, and the final sample comprised 126 students who completed the study measures. The inclusion criteria required that students be within the specified age range and enrolled at the college. Students with any psychological disorders were excluded to ensure the sample’s suitability for examining self-efficacy and glossophobia-related factors.

### 2.3 Instruments

#### 2.3.1 Glossophobia scale

We used the Glossophobia scale developed by Al-Ashqar (40). This scale was selected because it was prepared in a Saudi environment. The scale consists of 40 phrases. Each phrase had five graded answers (always, often, sometimes, rarely, never), and answers took the following degrees in order (four, three, two, one, zero). The scale included eight negative statements (4-8 - 16 - 20 - 24 - 28 - 36 - 40) with the opposite of the previous correction. To verify the reliability of this scale, Al-Ashqar (40) re-applied the scale to a sample of 136 students with an appropriate time difference. The correlation ratio between the two applications was 0.94. Using Cronbach’s alpha, the value reached (0.95), which is acceptable for appropriate reliability. Al-Ashqar (40) stated that the scale’s validity was confirmed through content validity. The scale was presented to (20) faculty members specializing in psychology from Saudi universities. All statements objected to by more than 85% of the referees were deleted.

#### 2.3.2 Self-efficacy scale

Radwan (41) translated it into the scale of (42). The scale has ten items, each asking the subject to choose an option from a graduated scale (no, rarely, often, and always). A low number indicates low general self-efficacy, whereas a high score indicates great general self-efficacy. The total score runs from 10 to 40. The application takes 3 to 7 minutes and can be performed individually or collectively. The scale was retested by Radwan (41) six weeks later. The participants were 37 students, and the reliability value was 0.71. Eid (43) retested the scale two weeks later with a sample consisting of 26 university students in Saudi Arabia. The reliability value was 0.75. Radwan (41) determined the criterion validity of this scale, and the correlation coefficient was 0.60, indicating that it has appropriate validity.

#### 2.3.3 Motivation scale

The motivation scale was prepared by Eid (44). It consisted of 36 phrases. Its participants indicate their degree of agreement on a Likert-type scale ranging from 1 (strongly disagree) to 7 (strongly agree) (strongly agree). The scale assesses inner motivation, identified regulation, and external regulation. Test-retest reliability was calculated to assess the consistency of this scale. The scale was administered to 32 college students in the Kingdom of Saudi Arabia and retested two weeks later. The reliability value was 0.89. Eid (44) stated that, to investigate the internal consistency, the results showed statistically significant correlations between item scores and the overall scale score, confirming the homogeneity of the statements and their high measurement capacity.

## 3 Results

### 3.1 Research question 1

To find out the result of this question, the researcher applied the Glossophobia Scale to a sample of (126) students.

To calculate the prevalence rate of glossophobia, the researcher determined the levels of the hypothesis test following Eid (43):

Range = Highest Score on Glossophobia Scale (160) - Lowest Score (0) = 160

Class length = 160 ÷ 5 (number of responses to the Glossophobia Scale) = 32

The score ranges corresponding to each response level on the Glossophobia Scale are presented in **Table 1**.

**Table 1:** Range of every response of the glossophobia scale.

Response	*Range of response*
Never	0–32
Rarely	32–64
Sometimes	64–96
Often	96–128
Always	128–160

As detailed in **Table 1**, the researcher selected a score of 96 as the cutoff threshold for the hypothesis, representing the lower boundary of the "Often" response category to distinguish between high and low levels of glossophobia (43).

**Table 2** makes it evident that there is a 22% prevalence of glossophobia among college students. According to research by Eid (6), 19.8% of male students in the Kingdom of Saudi Arabia have a speech anxiety disorder. According to Bartholomay and Houlihan’s (7) research, public speaking anxiety (PSA) affects roughly one in five people, which implies they estimated that glossophobia is 20 per cent common. The prevalence rate of glossophobia in the current study (22%) is lower than that reported by Pratiwi et al. (5), who found that 77.42% of students experienced speaking difficulties, but it is highly consistent with the findings of Bartholomay and Houlihan (7), who estimated the prevalence at approximately 20%. The current study’s prevalence rate of glossophobia among university students indicates that it is a widespread problem. This idea is supported by Hau Tse (18), who claimed that glossophobia is common.

**Table 2:** The prevalence of glossophobia in university students.

Variable	*N*	Number of students with high levels of *Glossophobia*	Percentage
Glossophobia	126	28	22%

Moreover, Goberman et al. (20) found unfavourable effects on interpersonal communication, academic performance, and career development. Therefore, this issue needs extra attention from researchers and educational institutions. Also, it could be helpful to introduce treatment with group counselling using different psychological techniques to control students’ glossophobia.

### 3.2 Research question 2

Pearson’s correlation was used in **Table 3** to examine the relationship between glossophobia and self-efficacy.

**Table 3:** Correlation between glossophobia and self-efficacy.

Correlation	*N*	Value	Sig.
Glossophobia and self-efficacy	126	-.300	.017

The result of the second question exhibited that there was a negative relationship between glossophobia and self-efficacy. Participants in the present study indicated that the total self-efficacy score was related to glossophobia. The results of the study by Qurnia and Marlina (36) demonstrated that the level of glossophobia correlates with self-efficacy. Prentiss (37) stated that self-efficacy is one of the best practices for improving speaking ability, and this could lead to the perspective that increasing self-efficacy may help reduce glossophobia. In terms of self-efficacy, research has shown that students with prior public speaking experience or training tend to have higher levels of self-efficacy than those without (45). Furthermore, students who receive positive feedback and support from their classmates and professors may have higher levels of self-efficacy (46).

According to the components of self-efficacy, one of the most important is self-regulation, which controls thoughts and could help control this disorder. Glossophobia requires the person to have the upper hand on their thoughts and emotions and acquire many strategies to manage their feelings of fear. Thus, acquiring some self-efficacy skills can help university students reduce their glossophobia.

### 3.3 Research question 3

To answer this question, we used Pearson’s correlation (**Table 4**) to examine the relationship between glossophobia and motivation.

**Table 4:** Correlation between glossophobia and motivation.

Correlation	*N*	Value	Sig.
Glossophobia and motivation	126	-.188	.035

The correlation value indicated a significant negative correlation between glossophobia and motivation. Saidalvi and Abdul Samad (47) found a relationship between motivational feedback and public speaking ability, which confirms the result of research question 2 of the present study: that is, if a student suffers from public speaking anxiety, his motivation will not be at the average level. Crawford et al. (38) found that individuals with high social anxiety experienced a reduction in social motivation during their interactions with others. The two previous studies showed that social interactions require motivation to drive individuals to participate effectively. On the other hand, glossophobia prohibits people from having a successful impact during important social meetings.

To illustrate the negative relationship between glossophobia and motivation, we can cite Stewart et al. (48), who defined motivation as “a driving force that encourages a person to engage in a task.” On the contrary, Hancock et al. (9) and Waheed (10) reported that PSA predicts a strong flight from public speaking due to embarrassment or shyness. So, motivation is a kind of encouragement to communicate with others, but glossophobia is a withdrawal to avoid others.

### 3.4 Research question 4

The findings are presented in **Table 5**, showing no relationship between glossophobia and age.

**Table 5:** Correlation between glossophobia and age.

Correlation	N	Value	Sig.
Glossophobia and age	126	-.08	.929

The results of the fourth question in the present study showed that there is no relationship between glossophobia and age, indicating that glossophobia does not become more severe with age. So, life’s irrational beliefs and feelings, whether in the young or the old, may play a greater role in manifesting glossophobia.

The result of research question 4 is like the finding of Eid (6): there was no statistically significant variance between the older and younger students with Speech Anxiety Disorder. Moreover, Khan et al. (24) found that fifth-year students had the lowest anxiety scores. Conversely, Gaibani and Elmenfi (26) found a substantial adverse effect of age on public speaking anxiety.

The current finding that age does not affect glossophobia is evident in the fact that a young person can give a better speech and speak with high self-confidence in front of a large audience, whereas an older person cannot do so in the same situation.

Mann–Whitney test was used in **Table 6** to answer this question, investigating the differences between male and female university students in Glossophobia.

**Table 6:** Mann–Whitney U test of glossophobia between males and females.

Variable	Gender	N	Mean rank	U	W	Z	Sig.
Glossophobia	Males	64	63.96	2, 013	4, 093	.144	.886
	Females	62	63.02				

The results showed no differences in glossophobia between male and female students. Male and female presenters were reported to experience varying degrees of public-speaking dread, according to Hancock et al. (9). Perveen et al. (25) and Khan et al. (24) found that female students exhibited a higher rate of glossophobia than male students. Also, the study by Waheed (10) showed that females have higher levels of glossophobia than males. The result of the study by Hau Tse (18) is similar to the result of the current question. Hence, gender does not increase glossophobia, but situations and the audience control the condition of the individual to have more or less fear of speaking.

There is conflicting information in the literature regarding gender differences in glossophobia, and it can be assumed that gender does not influence it. The author believed that high or low glossophobia depends mainly on the person’s willingness to believe their irrational thoughts about their ability to speak in front of an audience and not on the person’s gender.

## 4 Discussion

### 4.1 Theoretical interpretation of glossophobia prevalence

The descriptive analysis revealed a 22% prevalence rate of glossophobia among the sampled university students. Rather than viewing The descriptive this statistical reality in isolation, it must be interpreted through the lens of Bandura’s Social Cognitive Theory (SCT). In an academic environment transitioning rapidly under Saudi Arabia’s Vision 2030, students are increasingly required to engage in high-stakes public presentations, interactive seminars, and competitive group assignments. Within this context, the 22% prevalence indicates that nearly one-in-five students experience debilitating emotional arousal when executing these communication tasks.

This frequency aligns closely with Bartholomay and Houlihan’s (7) baseline of 20%, but stands in contrast to the much higher behavioural avoidance (77.42%) reported by Pratiwi et al. (5). The divergence suggests that while the vast majority of students may dislike or feel uneasy about public self-presentation, 22% suffer from clinical or subclinical glossophobia—a state driven by severe cognitive biases in which the cost of perceived failure is magnified. This structural anxiety requires targeted, institutional psychological interventions, moving beyond generic academic coaching to structured self-efficacy and anxiety management workshops.

### 4.2 Cognitive-behavioral mechanisms behind the self-efficacy and glossophobia link

The Pearson correlation analysis demonstrated that self-efficacy is a powerful, significant negative predictor of glossophobia. From a cognitive-behavioural perspective, this relationship highlights the exact *self-regulatory mechanisms* through which self-appraisals govern affective states. Self-efficacy operates as a cognitive filter; students with robust self-efficacy judge public speaking not as a threat to their social identity, but as a manageable challenge or a mastery experience. Consequently, they deploy adaptive coping strategies, maintain focus on task execution, and minimise internal distractions.

Conversely, low self-efficacy triggers a cascade of negative cognitive mechanisms, primarily cognitive interference and catastrophizing. When a student has weak beliefs in their speaking abilities, their working memory becomes flooded with self-doubt, anticipated humiliation, and hyper-awareness of the audience’s perceived critical evaluation. This cognitive clutter directly activates the autonomic nervous system, manifesting as the *somatic symptoms* noted in glossophobia—such as elevated heart rate, voice tremors, sweating, and memory blanks.

The student misinterprets these physiological sensations as definitive proof of impending failure, creating a vicious feedback loop: negative self-appraisals exacerbate somatic anxiety, which in turn reinforces the belief that one is incompetent. This explains why building mastery experiences and verbal persuasion (the structural pillars of self-efficacy) are critical psychological interventions to break the cycle of speaking anxiety.

### 4.3 Impact of cognitive and emotional states on behavioural drive (motivation)

The negative predictive relationship between glossophobia and academic motivation highlights the behavioural consequences of emotional distress within the SCT framework. Motivation is inherently dynamic and relies heavily on a student’s perceived probability of success and psychological safety. When a student is gripped by glossophobia, the anticipated psychological cost of participating in class discussions or giving presentations becomes overwhelming.

The underlying psychological mechanism here is defensive avoidance: to protect their self-esteem from public embarrassment, students proactively withdraw their psychological and behavioural investment. This emotional barrier severely dampens intrinsic motivation, transforming an assignment that could offer a sense of autonomy and learning into an existential threat. This explains the findings of Crawford et al. (38), where heightened social anxiety systematically diminished social and academic motivation. When glossophobia dominates the emotional climate, a student’s internal driving force shifts from achievement orientation to failure avoidance, ultimately impairing long-term academic engagement and professional development.

### 4.4 Socio-cultural moderation of psychological mechanisms (age and gender)

The statistical models revealed no significant differences in glossophobia across gender or age within this sample, challenging several traditional Western and regional findings. In previous literature, such as Khan et al. (24) and Perveen et al. (25), female students consistently exhibited significantly higher rates of public speaking anxiety due to varied socialisation patterns. However, our findings show a gender-neutral vulnerability to glossophobia among Saudi university students.

This lack of demographic variance can be explained by overarching cultural norms regarding public self-presentation in the region. In the contemporary Saudi academic context, both male and female undergraduates face identical, highly rigorous expectations to perform, present, and defend their work publicly as part of modern educational standards. The psychological pressure to maintain collective social face and meet high familial expectations operates uniformly across both genders.

Furthermore, the absence of an age or academic-year effect suggests that glossophobia is fundamentally a cognitive-developmental issue rather than a chronological one. Public speaking anxiety does not naturally decay with age or simple exposure; if the underlying cognitive distortions (such as catastrophizing and low self-efficacy) are not actively treated through deliberate cognitive restructuring or counselling interventions, the psychological vulnerability persists regardless of whether a student is a freshman or a senior.

## 5 Limitations of the study

The study may only contain a subset of university students, who may not reflect other age groups or communities with varying educational levels. The study can only demonstrate correlations between glossophobia, self-efficacy, and demographic characteristics; no correlation or directionality can be established.

## 6 Conclusion

The study aimed to investigate glossophobia, self-efficacy, and motivation among university students. The current study’s outcomes provide universities, researchers, and faculty members with up-to-date data that some students are more interested in. Since there is a negative correlation between glossophobia and motivation, it is evident that university students with glossophobia should have access to counselling programs to teach them strategies to increase their motivation. In addition, it is necessary to observe university students’ interaction with peers in different social situations. It is crucial to measure motivation and development in university students’ learning. Counselling and support programmes may provide students with strategies to manage public speaking anxiety and may support their engagement in academic and professional activities. The findings of Lihui (28) and Nelson et al. (27) support the effectiveness of counselling programs in reducing glossophobia.

According to the results of the second question, there is a significant correlation between glossophobia and self-efficacy; this suggests that students with higher self-efficacy tended to report lower levels of glossophobia. These findings highlight the potential relevance of self-efficacy and motivation in understanding students’ experiences of glossophobia. This would support the need for more observation, assessment, and counselling programs to better support students in improving their psychological conditions to control glossophobia. Hence, they will be more encouraged to participate in learning routine activities to improve academic performance.

The results of the current study demonstrated no statistically significant differences in glossophobia levels between male and female university students in the Kingdom of Saudi Arabia. This suggests that the psychological distress associated with public speaking affects both genders similarly within this academic context. Consequently, gender-neutral support systems and confidence-building workshops should be equally accessible to all students to mitigate speaking anxiety effectively.

The differences in glossophobia between male and female university students have not been validated. Most of the literature (E.g., 24, 25) claims that females have more glossophobia than males. Conversely, the result of the fourth question concludes that male and female university students in the Kingdom of Saudi Arabia have glossophobia to almost the same extent. The results of the present study showed no correlation between glossophobia and age, suggesting that age does not influence glossophobia. The result claims that younger individuals may have more glossophobia than older individuals, which is a condition that is unrelated to age.

Finally, glossophobia, or the dread of public speaking, is a widespread problem that affects many people. Social anxiety and low self-esteem are two psychological elements that may contribute to the development of glossophobia. It is apparent that glossophobia can have a considerable influence on an individual’s academic, professional, and personal life, as public speaking is an essential ability in many disciplines. Individuals can overcome their fear and enhance their public speaking skills by addressing the underlying issues that contribute to glossophobia. Practicing self-efficacy and requesting help from peers or professionals. As a result, it is critical for people to identify the causes of their fear of public speaking and take action to overcome it, allowing them to fulfil their potential in both their personal and professional lives.

### 6.1 Pedagogical implications

Since the outcomes of the current research showed that the occurrence of glossophobia among university students reached 22%, which is higher than the prevalence rate found in the studies of (6, 7), it was clear that Saudi universities should pay more attention to students who have glossophobia. This can help them solve academic problems requiring public speaking skills. Also, increasing motivation can be a source of support and encouragement to assist students who have glossophobia in eliminating their irrational fears that negatively affect their academic performance.

### 6.2 Recommendations

According to the counselling centres in different universities, it is recommended to introduce public counselling and instructional meetings to provide the students with basic information about the nature of glossophobia and the best techniques to overcome their fears. Increase the generalizability of the findings by expanding the study sample to encompass a broader range of demographic factors, such as culture, ethnicity, and socioeconomic status. Moreover, it would be helpful to provide the students with a high glossophobia with appropriate interventions to help them understand illogical thoughts and negative feelings related to this disorder. This could be beneficial to control their glossophobia. Consider researching the efficacy of interventions such as cognitive-behavioural therapy or exposure therapy in reducing glossophobia and improving psychological outcomes.

### 6.3 Future research

The current study used a sample of university students, so it would be valuable if there were a future study concerning the glossophobia of teenage students in high schools. The present study’s findings revealed a detrimental relationship between motivation and glossophobia, so it would be helpful to conduct a counselling program to reduce glossophobia in a future study. This could help to study the effectiveness of reducing glossophobia on motivation, which can lead to better academic performance. It would also be useful to examine the relationship between glossophobia and low or high academic achievement.

## Data Availability

The original contributions presented in the study are included in the article/supplementary material. Further inquiries can be directed to the corresponding author.
